# Epileptic crisis in a patient with obstructive sleep apnea during drug-induced endoscopy

**DOI:** 10.5935/1984-0063.20200123

**Published:** 2021

**Authors:** Laura Rodríguez-Alcalá, Carlos O´connor Reina, Blas Rodríguez Gil, Guillermo Plaza, Juan Carlos Casado Morente

**Affiliations:** 1 Department of Otorhinolaryngology. Hospital San Cecilio - Granada - Spain.; 2 Department of Otorhinolaryngology. Hospital Quiron Salud Marbella & Hospital Quiron Salud Campo de Gibraltar. Spain.; 3 Department of Anesthesiology. Hospital Quiron Salud Marbella. Spain; 4 Department of Otorhinolaryngology. Hospital Sanitas La Zarzuela & Hospital Universitario Fuenlabrada. Universidad Rey Juan Carlos. Madrid. Spain.

**Keywords:** Drug-induced Sleep Endoscopy, Propofol, Seizures

## Abstract

Drug-induced sleep endoscopy (DISE) is a complementary method for the diagnosis of obstruction sites in patients with snoring and obstructive sleep apnea (OSA) and allows the optimization of treatment. We present the first case of a patient having a seizure during DISE, after sedation with midazolam and propofol. We recommend that DISE should be performed in a safe environment, under monitoring, and with anesthesia equipment that can be used to counteract potential complications caused by seizures.

## INTRODUCTION

Since its appearance in the 1970s, propofol has become the IV hypnotic most used today. Imperial Chemical Industries developed this drug in the UK as ICI 35868 from studies of the sedative properties of phenolic derivatives in mice. Propofol is indicated for the induction and maintenance of anesthesia and sedation both inside and outside the operating room.

DISE consists of the induction of a short sleep (less than 20 minutes) using drugs that allow the results obtained to be reliably assimilated to natural sleep. To date, DISE is considered a very safe test and practically free of side effects.

## CASE REPORT

A 42-year-old man was diagnosed with OSA and had an apnea-hypopnea index of 31.4. He did not tolerate continuous positive airway pressure therapy. Among his pathology, type I obesity and hypertension treated with diet and one antihypertensive. No other personal or family history of interest.

Examination showed a lateral collapse during the Müller maneuver, palatal hypertrophy, and Friedman lingual tonsil hypertrophy stage 3. Drug-induced sleep endoscopy (DISE) was proposed to evaluate the site of obstruction during sleep, and the patient agreed and gave informed consent. The pre-anesthetic assessment revealed no known allergies or significant medical antecedents. Examination showed a Mallampati I and the preoperative state was evaluated as ASA I (American Society of Anesthesiologist). The DISE protocol consists of monitoring the level of anesthetic depth and neurological monitoring using BIS (bispectral index). Sedation is performed by propofol pump (2-4mg/kg/h) if possible (if not available, it is performed by propofol boluses) with the aim of maintaining a BIS between 50 and 70. The duration of the procedure It is approximately 15-20min when we observe snoring and three apnea pauses with the PaO_2_<90%. Monitoring of PaCO_2_ with capnography is recommended, but not mandatory, as it is intended that the patient does not lose spontaneous ventilation. Induction of physiological sleep is achieved with a Ramsay 4-5 (moderate level of sedation)^1^.

In our case we carried out the DISE, showed pronounced basilingual and soft palate hypertrophy that were both responsible for the airway collapse. For sedation, in this case, 0,02mg/kg dose of midazolam and propofol boluses (1-1.5mg/kg) and maintenance with boluses of 20mg every 3-5 minutes according to sedation needs. A total dose of 250 mg was used. The procedure was performed without adverse incidents until an anomalous reaction occurred during the awakening. The patient adopted a decerebrate and unconscious position that did not revert after intravenous flumazenil administration. An orotracheal tube was placed and passed to control mechanical ventilation with FiO_2_ 60-80%.

**Figure 1:**
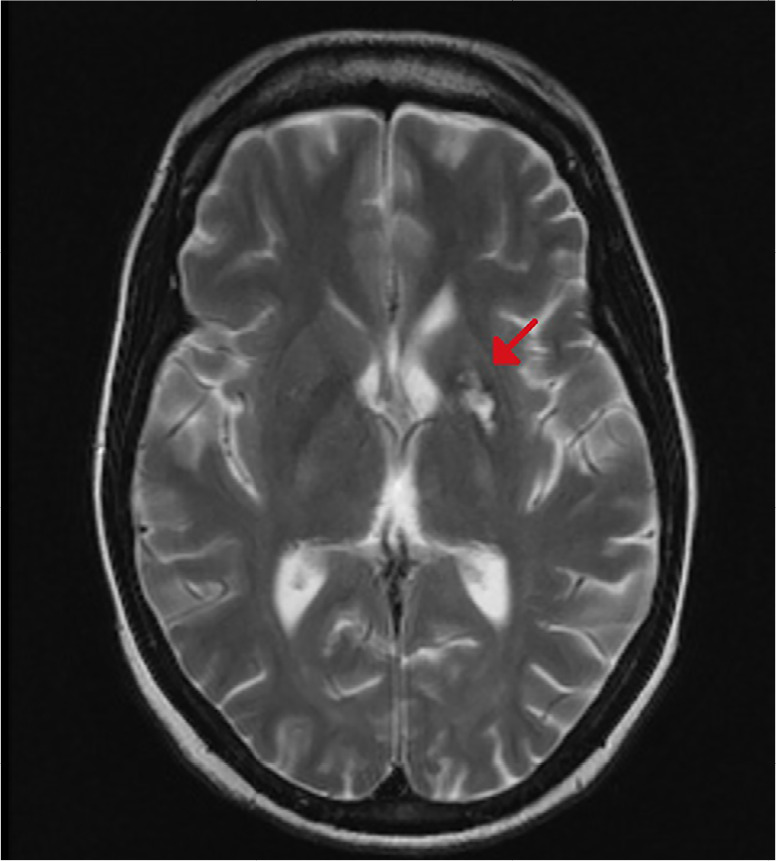
MRI showed no significant pathology once patient awake.

**Figure 2:**
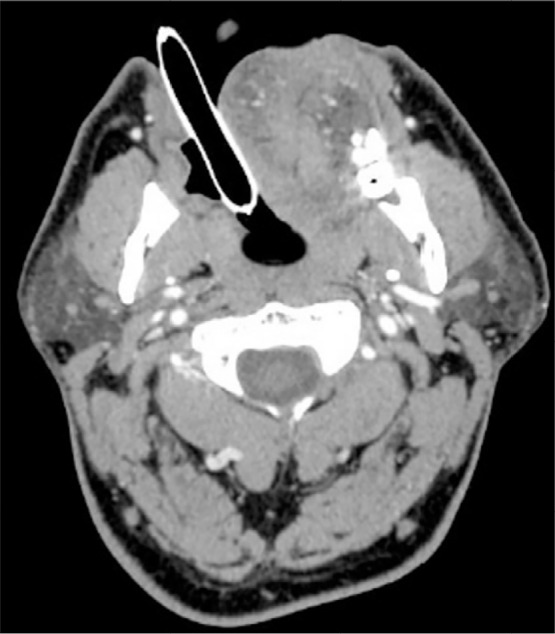
CT scan performed with patient intubated and assisted ventilation.

**Figure 3:**
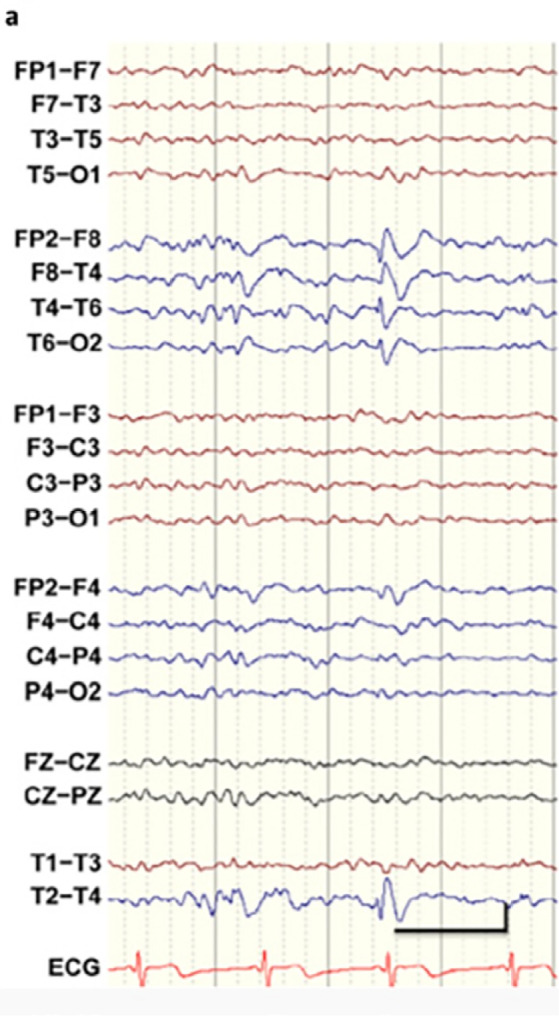
EEG showed an epileptiform tip in the right temporal region (phase inversion in T4). The tips are normally accompanied by a slow wave behind and are specific to epilepsy. In this case, the patient had temporary epilepsy.

During intensive care, an urgent computed tomography (CT) scan was performed. Assessment by neurologist on duty was considered compatible with the seizure episode in relation to the epileptogenic activity of propofol. Levetiracetam was administered. After the patient had been extubated, he was sent to the intensive care unit for monitoring. No significant alterations in the internal, vertebral, or basilar carotid artery or at the level of the polygon of Willis were observed in the cranial CT scan. (Figure 2) Neurological examination revealed no meningeal signs. The cranial nerves were normal, and reflexes were also normal and symmetrical. All blood tests were normal. Magnetic resonance imaging (MRI) was performed 12 hours after the epileptic episode and showed some nonspecific lesions in the subcortical white matter, reported by the radiologist as chronic lesions (chronic ischemia). There were no foci of restriction in the parenchyma or mass effect. The ventricular system and structures of the cranial base had normal size and morphology. (Figure 1) The EEG in the intercritical period (<12h) showed epilectogenic activity (Figure 3). However, at 72h, the electrophysiological test showed no epilectogenic activity.

## DISCUSSION

DISE is a very useful tool for the diagnosis of OSA. However, it must be performed in a safe clinical setting, a clinical operating or endoscopy room with standard anesthesia equipment for monitoring and resuscitation in case of emergency^2^. The precipitating cause of the seizure during DISE is uncertain. Hypoxemia is a common metabolic cause of seizures^3,4^. Patients with OSA have episodes of anoxia and hypercapnia secondary to the imbalance between the caliber of the airway and the motor tone of the tongue, or of the dilator muscles of the airways^5,6^. Because of the lack of adequate alveolar ventilation, resulting from the narrowing of the upper airway, oxygen saturation may fall and the partial pressure of CO_2_ may increase^7^. Another precipitating cause of the seizure in our case might have been the medications administered during anesthesia.

Propofol is recommended as a sedative during DISE; however, it can be questioned in patients with significant desaturations during the polysomnographic study. Propofol carries out its hypnotic action primarily by potentiating the chlorine current induced by g-aminobutyric acid (GABA) through its binding to the b subunit of the GABA receptor. The sites of the b1, b2 and b3 subunits of the transmembrane domains play a key role in the hypnotic action of propofol^8,9^. The onset of hypnosis after administration of a dose of 2.5mg/kg is rapid (arm-brain circulation) and reaches a maximum effect after 90 to 100 s. The median effective dose (ED50) of propofol for unconsciousness is between 1 and 1.5mg/kg for bolus administration. The duration of hypnosis depends on the dose and is between 5 and 10 minidosis of 2 to 2.5mg/kg^10^. Although, the effect of propofol on epileptogenic EEG activity has raised some controversy. It could suppress seizure activity through GABA agonism, inhibition of NMDA receptors (RNMDA), and modulation of slow calcium ion channels. However, this agonism with GABA and antagonism with glycine could induce clinical seizures and epileptiform alterations in the EEG^11^, especially during the induction of anesthesia and awakening.

Propofol exerts a dose-dependent anticonvulsant action. Propofol has been used in the treatment of epileptic seizures. However, it can cause generalized tonic-clonic seizures and has been used in the cortical mapping of epileptogenic foci^12^. Propofol and midazolam were used in this patient up to the time the seizure occurred. Only propofol has been shown to induce convulsions, or manifestations similar to those of seizures. Although DISE is not the gold standard in the diagnosis of obstructive sleep apnea, it is currently the test that allows locating sites of obstruction in conditions that best approximate natural sleep. Without carrying it out, the expected benefit may not be obtained with the treatment proposed by the sleep surgeon. It achieves a better and safer assessment of the most appropriate treatment option for each subject suffering from OSA, which in turn increases the chances of success. It is very useful to avoid more risky and costly procedures without guaranteeing a certain benefit for the patient^13^.
